# A Fast Multi-Locus Ridge Regression Algorithm for High-Dimensional Genome-Wide Association Studies

**DOI:** 10.3389/fgene.2021.649196

**Published:** 2021-03-29

**Authors:** Jin Zhang, Min Chen, Yangjun Wen, Yin Zhang, Yunan Lu, Shengmeng Wang, Juncong Chen

**Affiliations:** ^1^College of Science, Nanjing Agricultural University, Nanjing, China; ^2^Postdoctoral Research Station of Crop Science, Nanjing Agricultural University, Nanjing, China; ^3^College of Finance, Nanjing Agricultural University, Nanjing, China

**Keywords:** genome-wide association study, mixed linear model, multi-locus algorithm, statistical power, polygenic background, minor effect

## Abstract

The mixed linear model (MLM) has been widely used in genome-wide association study (GWAS) to dissect quantitative traits in human, animal, and plant genetics. Most methodologies consider all single nucleotide polymorphism (SNP) effects as random effects under the MLM framework, which fail to detect the joint minor effect of multiple genetic markers on a trait. Therefore, polygenes with minor effects remain largely unexplored in today’s big data era. In this study, we developed a new algorithm under the MLM framework, which is called the fast multi-locus ridge regression (FastRR) algorithm. The FastRR algorithm first whitens the covariance matrix of the polygenic matrix K and environmental noise, then selects potentially related SNPs among large scale markers, which have a high correlation with the target trait, and finally analyzes the subset variables using a multi-locus deshrinking ridge regression for true quantitative trait nucleotide (QTN) detection. Results from the analyses of both simulated and real data show that the FastRR algorithm is more powerful for both large and small QTN detection, more accurate in QTN effect estimation, and has more stable results under various polygenic backgrounds. Moreover, compared with existing methods, the FastRR algorithm has the advantage of high computing speed. In conclusion, the FastRR algorithm provides an alternative algorithm for multi-locus GWAS in high dimensional genomic datasets.

## Introduction

Genome-wide association study (GWAS) has been widely used in the genetic dissection of quantitative traits in human, animal, and plant genetics. GWAS typically searches for the correlations between genetic variants and hundreds or thousands of individuals. However, a complete characterization of the biological mechanism for most quantitative traits remains elusive (Dahl et al., 2016) and a number of polygenes with minor effects are unexplored (Zhang and Xu, 2005; Wen et al., 2019). This may be because the GWAS approach is still quite crude, and most of the minor biological associations between sequence and phenotype remain unmeasured. Recently, advanced biotechnology has generated large-scale single nucleotide polymorphisms (SNPs) and phenotypes, which have been valuable for genetic analysis. A large number of statistical methodologies for GWAS have been proposed (Atwell et al., 2010; Lippert et al., 2011; Zhou and Stephens, 2012; Wen et al., 2018, 2020; Sun et al., 2019; Wang et al., 2020).

Since the introduction of the Q + K (Q represents the population structure and K represents the kinship matrix) mixed linear model (MLM) approach (Yu et al., 2006) to the concept of GWAS, the power of quantitative trait nucleotide (QTN) detection has been significantly increased. On this basis, the compressed MLM (Zhang et al., 2010) and enriched compressed MLM (Li et al., 2014) have been proposed to improve computational efficiency. Meanwhile, an efficient mixed model association (EMMA) (Kang et al., 2008) was regarded as the milestone improvement in the MLM approach, which treated the polygenic effect as the random effect to fit the mixed model. Currently, this concept has become more and more popular in genomic analysis. A number of methods based on this concept are continually emerging, such as EMMAX (Kang et al., 2010), FaST-LMM (Lippert et al., 2011), and GEMMA (Zhou and Stephens, 2012). Because of the dissection of genetic variants and computational speed, all these methods have been successfully applied in MLM. For all the above methods, they comprise a one-dimensional genome scan by testing one marker at a time, more importantly, the SNP effect is considered as the fixed effect, which may be disadvantageous to the detection of QTN in GWAS (Goddard et al., 2009; Zhang et al., 2017; Wen et al., 2018, 2020).

Although the current single variant methods of GWAS have succeeded in identifying QTNs associated with the interested traits, these approaches fail to consider the joint minor effect of multiple genetic markers on a trait (Tamba et al., 2017); furthermore, they do not match the internal genetic mechanism of these quantitative traits (Tamba et al., 2017; Zhang et al., 2017; Sun et al., 2019; Wen et al., 2019). To overcome this drawback, multi-locus methodologies have been developed, such as least absolute shrinkage and selection operator (lasso) (Tibshirani, 1996; Xu, 2010; Zhang et al., 2012), Bayesian lasso (Yi and Xu, 2008), adaptive mixed lasso (Wang et al., 2011), and empirical Bayes (Xu, 2007). All SNPs can be included in the model and can be simultaneously estimated by using multi-locus methodologies. If the number of SNPs (*p*) is many times larger than the number of individuals (*n*), the approaches will fail to analyze this oversaturated model. Under this circumstance, a natural response is to consider reducing the number of SNP effects in the multi-locus genetic model. Zhou et al. (2013) and Moser et al. (2015) proposed the Bayesian model, which estimates only a few variance components instead of considering all. It is an alternative approach to solve the “big *p*, small *n*” problem. Currently, two-stage methodologies (Tamba et al., 2017; Zhang et al., 2017; Wen et al., 2018) borrowed this idea and have been proposed for multi-locus GWAS. All these methodologies provide the tools for high-dimensional genetic data analysis. It is known that the quantitative traits are controlled by a few genes with large effects and numerous polygenes with minor effects. Nevertheless, the dissection of the polygenes with minor effects needs to be improved in above mentioned multi-locus approaches.

In this study, we propose a multi-stage flexible approach for GWAS to detect the associated (large and minor effects) variables/SNPs. In our model, the fast multi-locus ridge regression algorithm (FastRR), all SNP effects are considered as random effects. The FastRR algorithm first whitens the covariance matrix of the polygenic matrix K and environmental noise. Subsequently, the FastRR algorithm reduces the number of SNPs according to correlation, the variables of which significantly correlate with the response are retained for the next stage. In the final stage, deshrinking ridge regression (DRR) is applied to implement parametric estimation and significance tests of variables. In this study, a series of simulated and real dataset analyses are used to validate this new method. For comparison, five established methods – lasso, adaptive lasso, smoothly clipped absolute deviation (SCAD), EMMA, and decontaminated efficient mixed model association (DEMMA) are used for analysis.

## Materials and Methods

### Genetic Model

Let *y*_*i*_(*i* = 1, 2, …, *n*) be the phenotypic value of the *i*-th individual in a sample of size *n* from a natural population, and the genetic model can be described as:

(1) y=Wα+Zγ+u+ε

where **y** = (*y*_1_, …, *y*_*n*_)^*T*^; ***α*** is a *c* × 1 vector of the fixed effects, such as the intercept, population structure effect and so on, **W** is the corresponding designed matrix for ***α***; **Z** is an *n* × 1 vector of marker genotypes, and $\gamma ∼N\left(0,\sigma_{\gamma }^{2}\right)$ is a random effect of putative QTN. $\sigma_{\gamma }^{2}$ is the variance of the putative QTN; u∼MVN(0,σg2K) is an *n* × 1 random vector of polygenic effects, σg2 is the variance of polygenic background, **K** is a known *n* × *n* relatedness matrix; ***ε*** is an *n* × 1 vector of residual errors with an assumed *MVN*(**0**, *σ*^2^**I**_*n*_) distribution; *σ*^2^ is the variance of residual error; and **I**_*n*_ is a *n* × *n* identity matrix. *MVN* denotes multivariate normal distribution.

As γ is treated as being a random effect, the variance of ***y*** in the model (1) is:

(2) var(y)=σγ2ZZT+σg2K+σ2In=σ2(λγZZT+λgK+In)

where λγ=σγ2/σ2, λg=σg2/σ2.

### Fast Multi-Locus Ridge Regression Algorithm

The FastRR algorithm is a multi-stage flexible approach for GWAS, which simultaneously implements estimation and testing to detect associated variables/SNPs. We describe it with the following stages:

#### The Polygenic and Residual Noise Whitening Stage

The key point of solving the model (1) is to estimate two ratios of variance components, *λ*_*γ*_ and *λ*_*g*_, which cause expensive computational burden. It is noted that polygenic variance is always larger than zero, while variance components for most SNPs are zero because these markers are not associated with the interested trait, which is *λ*_*γ*_ = 0 for most SNPs. Therefore, in the first step, we estimate ${\hat{\lambda }}_{g}$ by the reduced form of the model (1), which deleted **Z***γ* with only polygenic background, and replace *λ*_*g*_ in (2) by the ${\hat{\lambda }}_{g}$ (Wen et al., 2018, 2020), avoiding re-estimate *λ*_*g*_ for each single marker scanning. Thus,

(3) $$var\left(y\right)=\sigma^{2}\left(\lambda_{\gamma }{\text{ZZ}}^{T}+{\hat{\lambda }}_{g}\text{K}+{\text{I}}_{n}\right)=\sigma^{2}\left(\lambda_{\gamma }{\text{ZZ}}^{T}+\text{B}\right)$$

An eigen (or spectral) decomposition of the positive definite matrix $\text{B}={\hat{\lambda }}_{g}\text{K}+{\text{I}}_{n}$ is:

(4) $$\text{B}=\text{Q}\Lambda {\text{Q}}^{T}=\left(\text{Q}\Lambda^{\frac{1}{2}}{\text{Q}}^{T}\right)\left(\text{Q}\Lambda^{\frac{1}{2}}{\text{Q}}^{T}\right)$$

where **Q** is orthogonal and **Λ** is a diagonal matrix with positive eigenvalues. Let $\text{C}=\text{Q}\Lambda^{-\frac{1}{2}}{\text{Q}}^{T}$, the model (1) is changed to:

(5) $${\text{y}}_{c}={\text{W}}_{c}\alpha +{\text{Z}}_{c}\gamma +\varepsilon_{c}$$

where, **y**_*c*_ = **Cy**, **W**_*c*_ = **CW**, **Z**_*c*_ = **CZ**, *ε*_*c*_ = **Cu**+**C***ε*∼*MVN*(**0**,*σ*^2^**I**_*n*_) (Wen et al., 2018, 2020).

#### Variable Reduction Stage

A number of studies have illustrated that most quantitative traits are controlled by a small portion of genes, including a few genes with large effects and polygenes with minor effects (Zhang et al., 2017; Wen et al., 2019). It is critical to dissect all associated loci from large-scale genetic markers. Herein, we conduct a variable reduction stage, whose purpose is dimension reduction. At this stage, the FastRR algorithm detects a subset of putative variables associated with the phenotype, and thus avoids the intractable computational problems of high-dimensional datasets analysis.

We calculate the marginal correlation coefficients between **Z**_*c*_ (variables after polygenic background correction) and **y**_*c*_ (phenotype after polygenic background correction) under model (5), R function *cor.test* returns the *p*-value of the correlation test. The critical value for significance was set at *p*-value < 0.01 (Tamba et al., 2017). For the threshold of 0.01, even the slight correlations between predictors and the response will be captured (Tamba et al., 2017), and the unassociated loci will be removed. All the most potential QTNs are selected to construct the reduced multi-locus model for the next stage. Essentially, this marginal correlation step is similar to the single marker scanning, which combined with the polygenic background without considering variance components $\sigma_{\gamma }^{2}$.

#### Parameter Estimation Stage

In the multi-locus model,

(6) y=Wα+Zγ+ε

where **y** is the phenotypic value of the quantitative trait, which is the same as that in the model (1); α is a vector of fixed effects, γ is a *q* × 1 random effect vector of the selected *q* markers from the above stage, and γ_*k*_ ∼ *N* (0, *ϕ*^2^), *k* = 1, …, *q*; **W** and **Z** are the corresponding design matrices for α and γ. Here, polygenic background correction is not considered in model (6), because the above two steps under the polygenic background model had already selected all potential associated QTNs. All the parameters in model (6) are estimated by DRR proposed by Wang et al. (2020).

Before introducing the DRR, let us briefly recall the ordinary ridge regression (ORR). According to the best linear unbiased prediction (BLUP) of the marker effects and the prediction error variances using the conditional expectation and conditional variance, the estimates of ORR are as follows,

(7) $${\hat{\gamma }}^{ORR}=E\left(\gamma |\text{y}\right)=\lambda {\text{Z}}^{T}{\text{H}}^{-1}\left(\text{y}-\text{W}\alpha \right)$$

(8) $$var\left({\hat{\gamma }}^{ORR}|\text{y}\right)=\left(\lambda \text{I}-\lambda {\text{Z}}^{T}{\text{H}}^{-1}\text{Z}\lambda \right)$$

where $\lambda =\frac{\phi^{2}}{\sigma^{2}},\text{H}=\left({\text{ZZ}}^{T}\right)\lambda +I_{n}$.

Ordinary ridge regression is inflexible and inaccurate for GWAS (Wang et al., 2020). Therefore, we apply the following DRR method, which can bring both the accurate effects and tests back. The essential difference between ORR and DRR is the well-measurement-factor (also called degree of freedom), which is

(9) dk=1-var(γ^kORR|y)ϕ2=λZkTH-1Zk

γ^kORR is the *k*-th element of ${\hat{\gamma }}^{ORR}$, where *ϕ*^2^and var(γ^kORR|y) are prior and posterior variances for γ_*k*_, respectively.

(10) γ^kDRR=ϕ2ϕ2-var(γ^kORR|y)γ^kORR=dk-1γ^kORR

(11) $$\begin{array}{c} var\left({\hat{\gamma }}_{k}^{DRR}\right)=\frac{\phi^{2}}{\phi^{2}-var\left({\hat{\gamma }}_{k}^{ORR}|\text{y}\right)}var\left({\hat{\gamma }}_{k}^{ORR}|\text{y}\right)\\ \;=d_{k}^{-1}var\left({\hat{\gamma }}_{k}^{ORR}|\text{y}\right) \end{array}$$

(12) $$W_{k}=\frac{{\left({\hat{\gamma }}_{k}^{DRR}\right)}^{2}}{var\left({\hat{\gamma }}_{k}^{DRR}\right)}=\frac{{\left({\hat{\gamma }}_{k}^{ORR}/d_{k}\right)}^{2}}{var\left({\hat{\gamma }}_{k}^{ORR}|\text{y}\right)/d_{k}}=d_{k}^{-1}\frac{{\left({\hat{\gamma }}_{k}^{ORR}\right)}^{2}}{var\left({\hat{\gamma }}_{k}^{ORR}|\text{y}\right)}$$

The test statistic of DRR, *W*_*k*_, follows a Chi-square distribution with one degree of freedom under the null model, *H*_0_:*γ*_*k*_ = 0. The DRR method deshrinks both the estimated effects of markers and their estimated variances from the ORR, resulting in deshrunk Wald test statistics.

### Comparison Methods

#### LASSO

Lasso regression (Tibshirani, 1996) is a type of linear regression that implements shrinkage by performing *L*_1_ regularization and selects the most correlated with response variables. It is a popular method for simultaneous estimation and variable selection. The method was implemented by the R software package *lars*^1^.

#### Adaptive Lasso

Similar to the lasso, the adaptive lasso (Zou, 2006) is a mainstream method of variable selection, in which the adaptive weights are used for penalizing different coefficients in the *L*_1_ penalty. Adaptive lasso shows more consistence for variable selection than lasso in data analysis. The method was implemented by the R software package *glmnet*^2^.

#### SCAD

SCAD (Fan and Li, 2001) as the variable selection has the nice oracle property. The estimator of SCAD attempts to alleviate bias from variable selection, while also retaining a continuous penalty that encourages sparsity. The method was implemented by the R software package *ncvreg*^3^.

#### EMMA

Efficient mixed-model association (Kang et al., 2008) is an established genome-wide single-marker scan methodology under the framework of MLM, in which the polygenic background and population structure are controlled. The method was implemented by the R software package EMMA^4^.

#### DEMMA

The polygenic effect (the sum of all marker effects) is treated as a random effect in EMMA. On the other side, EMMA already included the marker effect as the fixed effect. Thus, there are two effects for each marker, which lead to a reduced power for testing. Wang et al. (2020) proposed DEMMA to overcome the above drawback. The method was implemented by the R code^5^.

### Experimental Materials

#### The Simulation Data

Three Monte Carlo simulation experiments were conducted to evaluate the performances of the FastRR algorithm and other methods. We generated genotypes according to the minor allele frequency (MAF) in the interval (0.1, 0.5) under Hardy–Weinberg equilibrium. The simulation datasets contained *n* = 2000 individuals with *p* = 10,000 genetic variants, which were generated with MLM. The total average was set at 10.0 and residual variance was set at 10.0. We considered three scenarios for each simulation, including two times polygenic background, five times polygenic background, and ten times polygenic background.

Only one QTN with a fixed position (Table 1) was simulated and placed on the SNPs with 0.1 heritability for the first simulation; five QTNs with fixed positions were assigned and placed on the SNPs for the second simulation, the heritabilities of the QTNs were set as 0.02, 0.05, 0.05, 0.08, and 0.10, respectively. Their positions and effects are listed in Tables 2A–C. For the third simulation experiment, we randomly selected 100 QTNs, and the sum contribution of QTNs to the total phenotypic variance was 0.5. Each simulation experiment was repeated 100 times. The power for each QTN was defined as the proportion of samples over the threshold to the total number of replicates (100), the criterion for lasso, adaptive lasso, and SCAD was set as LOD ≥ 3.0, the criterion for ORR, EMMA, DEMMA, and the FastRR algorithm was set as 0.05/*p*, where *p* was the number of markers in the genetic model. The false positive rate was calculated as the ratio of the number of false positive effects to the total number of zero effects.

**TABLE 1:** Comparison of lasso, adaptive lasso, SCAD, EMMA, DEMMA, and FastRR methods in the first simulation experiment (three scenarios).

Polygenic back ground	True value			Lasso			Adaptive lasso			SCAD			EMMA			DEMMA			FastRR		
	Position	Effect	*r*^2^	Power (%)	Effect (SD)	MSE	Power (%)	Effect (SD)	MSE	Power (%)	Effect (SD)	MSE	Power (%)	Effect (SD)	MSE	Power (%)	Effect (SD)	MSE	Power (%)	Effect (SD)	MSE
2K	98	0.7398	5%	100.0	0.476 (0.092)	7.768	83.0	0.374 (0.155)	13.079	100.0	0.474 (0.156)	9.446	100.0	0.736 (0.091)	0.818	100.0	0.736 (0.091)	0.818	100.0	0.734 (0.091)	0.817
5K	98	0.7398	5%	100.0	0.404 (0.111)	12.527	59.0	0.315 (0.224)	13.585	100.0	0.390 (0.164)	14.915	98.0	0.735 (0.103)	1.040	99.0	0.733 (0.105)	1.089	100.0	0.729 (0.109)	1.188
10K	98	0.7398	5%	91.0	0.337 (0.134)	16.386	32.0	0.380 (0.247)	6.048	87.0	0.324 (0.168)	17.446	70.0	0.795 (0.094)	0.829	84.0	0.765 (0.110)	1.052	99.0	0.729 (0.131)	1.693
False positive rate of 2K (‰)				0.453			0.004			0.288			0.030			0.014			0.450		
False positive rate of 5K (‰)				0.555			0.001			0.460			0.090			0.018			0.498		
False positive rate of 10K (‰)				0.636			0.019			0.550			0.050			0.026			0.436		

*Three scenarios, including two times polygenic background, five times polygenic background, and ten times polygenic background. MSE, mean squared error. The numbers in parentheses represent the standard deviation.*

**TABLE 2A:** Comparison of lasso, adaptive lasso, SCAD, EMMA, DEMMA, and FastRR methods in the second simulation experiment (scenarios 1: two times polygenic background).

QTN	True value			Lasso			Adaptive lasso			SCAD			EMMA			DEMMA			FastRR		
	Position	Effect	*r*^2^	Power (%)	Effect (SD)	MSE	Power (%)	Effect (SD)	MSE	Power (%)	Effect (SD)	MSE	Power (%)	Effect (SD)	MSE	Power (%)	Effect (SD)	MSE	Power (%)	Effect (SD)	MSE
**Polygenic background (2K)**																					
1	98	0.5451	2%	99.0	0.298 (0.091)	6.833	96.0	0.416 (0.149)	3.703	99.0	0.269 (0.122)	9.011	91.0	0.600 (0.087)	0.956	94.0	0.596 (0.089)	0.978	99.0	0.587 (0.094)	1.035
2	301	0.8622	5%	100.0	0578 (0.100)	9.080	100.0	0.782 (0.114)	1.924	100.0	0.683 (0.174)	6.221	100.0	0.822 (0.095)	1.044	100.0	0.822 (0.095)	1.044	100.0	0.820 (0.094)	1.054
3	540	0.8598	5%	100.0	0.605 (0.093)	7.350	100.0	0.811 (0.101)	1.240	100.0	0.730 (0.150)	3.906	100.0	0.852 (0.089)	0.788	100.0	0.852 (0.089)	0.788	100.0	0.850 (0.089)	0.788
4	801	1.0789	8%	100.0	0.807 (0.099)	8.34	100.0	1.030 (0.105)	1.333	100.0	1.025 (0.139)	2.211	100.0	1.061 (0.094)	0.914	100.0	1.061 (0.094)	0.914	100.0	1.059 (0.094)	0.911
5	1000	1.2093	10%	100.0	0.957 (0.095)	7.276	100.0	1.118 (0.098)	1.023	100.0	1.207 (0.251)	10.129	100.0	1.223 (0.094)	0.886	100.0	1.223 (0.094)	0.886	100.0	1.220 (0.094)	0.878
False positive rate (‰)				0.461			0.024			0.355			0.000			0.007			0.422		

*Three scenarios, including two times polygenic background, five times polygenic background, and ten times polygenic background. MSE, mean squared error. The numbers in parentheses represent the standard deviation.*

**TABLE 2B:** Comparison of lasso, adaptive lasso, SCAD, EMMA, DEMMA, and FastRR methods in the second simulation experiment (scenarios 2: five times polygenic background)

QTN	True value			Lasso			Adaptive lasso			SCAD			EMMA			DEMMA			FastRR		
	Position	Effect	*r*^2^	Power (%)	Effect (SD)	MSE	Power (%)	Effect (SD)	MSE	Power (%)	Effect (SD)	MSE	Power (%)	Effect (SD)	MSE	Power (%)	Effect (SD)	MSE	Power (%)	Effect (SD)	MSE
**Polygenic background (5K)**																					
1	98	0.5451	2%	89.0	0.239 (0.091)	9.048	71.0	0.375 (0.179)	4.297	88.0	0.216 (0.098)	10.367	52.0	0.656 (0.072)	0.943	73.0	0.622 (0.082)	0.910	96.0	0.587 (0.095)	1.029
2	301	0.8622	5%	100.0	0.527 (0.119)	12.673	100.0	0.764 (0.166)	3.703	100.0	0.606 (0.200)	10.515	99.0	0.841 (0.106)	1.140	99.0	0.841 (0.106)	1.140	100.0	0.820 (0.126)	1.283
3	540	0.8598	5%	100.0	0.518 (0.117)	13.063	100.0	0.754 (0.153)	3.439	100.0	0.591 (0.191)	10.812	99.0	0.831 (0.107)	1.195	100.0	0.828 (0.110)	1.297	100.0	0.826 (0.109)	1.299
4	801	1.0789	8%	100.0	0.755 (0.116)	11.824	100.0	1.029 (0.126)	1.811	100.0	0.957 (0.186)	4.911	100.0	1.077 (0.117)	1.336	100.0	1.077 (0.116)	1.336	100.0	1.075 (0.116)	1.334
5	1000	1.2093	10%	100.0	0.897 (0.109)	10.937	100.0	1.176 (0.117)	1.480	100.0	1.165 (0.150)	2.428	100.0	1.234 (0.101)	1.063	100.0	1.234 (0.101)	1.063	100.0	1.232 (0.100)	1.049
False positive rate (‰)				0.510			0.102			0.473			0.040			0.014			0.431		

*Three scenarios, including two times polygenic background, five times polygenic background, and ten times polygenic background. MSE, mean squared error. The numbers in parentheses represent the standard deviation.*

**TABLE 2C:** Comparison of lasso, adaptive lasso, SCAD, EMMA, DEMMA, and FastRR methods in the second simulation experiment (scenarios 3: ten times polygenic background).

QTN	True value			Lasso			Adaptive lasso			SCAD			EMMA			DEMMA			FastRR		
	Position	Effect	*r*^2^	Power (%)	Effect (SD)	MSE	Power (%)	Effect (SD)	MSE	Power (%)	Effect (SD)	MSE	Power (%)	Effect (SD)	MSE	Power (%)	Effect (SD)	MSE	Power (%)	Effect (SD)	MSE
**Polygenic background (10K)**																					
1	98	0.5451	2%	56.0	0.223 (0.092)	6.283	46.0	0.393 (0.188)	4.297	51.0	0.240 (0.092)	5.165	20.0	0.757 (0.047)	0.943	36.0	0.706 (0.069)	1.102	76.0	0.644 (0.095)	1.160
2	301	0.8622	5%	97.0	0.437 (0.126)	19.080	93.0	0.718 (0.212)	6.046	98.0	0.488 (0.195)	17.444	89.0	0.860 (0.102)	0.923	93.0	0.851 (0.108)	1.088	100.0	0.830 (0.126)	1.668
3	540	0.8598	5%	97.0	0.459 (0.141)	17.520	97.0	0.726 (0.235)	1.240	98.0	0.516 (0.210)	15.874	88.0	0.873 (0.119)	1.242	94.0	0.858 (0.128)	1.529	99.0	0.842 (0.140)	1.960
4	801	1.0789	8%	100.0	0.682 (0.147)	17.912	99.0	1.020 (0.173)	3.287	100.0	0.855 (0.251)	11.254	100.0	1.085 (0.141)	1.962	100.0	1.085 (0.141)	1.962	100.0	1.083 (0.141)	1.958
5	1000	1.2093	10%	100.0	0.783 (0.159)	20.627	99.0	1.129 (0.174)	3.592	100.0	1.012 (0.251)	10.129	100.0	1.206 (0.152)	2.297	100	1.206 (0.153)	2.297	100.0	1.204 (0.152)	2.290
False positive rate (‰)				0.673			0.209			0.788			0.050			0.026			0.490		

*Three scenarios, including two times, five times and ten times the polygenic background. MSE, mean squared error. The numbers in parentheses represent the standard deviation.*

#### The Rice Data

To validate the FastRR algorithm, the rice data that was used in this study for GWAS demonstration consists of 524 inbred varieties, which were collected from China and southeast Asia (Chen et al., 2014; Wei et al., 2018). A total of 6.5 million high-quality SNPs covering 90% of total SNPs were analyzed by Chen et al. (2014). A total of 314,393 SNPs and grain width traits (Wang et al., 2020) were analyzed in this study. These data were downloaded from the link.^6^

#### The *Arabidopsis* Data

To further evaluate the performance of FastRR, we reanalyzed the genetic data sets of *Arabidopsis* published by Atwell et al. (2010). Both phenotypes and genotypes were obtained from the link^7^. A total of 199 *Arabidopsis* lines and 216,130 SNPs were used for analysis. Among all traits, we analyzed three traits related to flowering time: (1) LD: days to flowering under long days; (2) SD: days to flowering under short days; and (3) SDV: days to flowering under short days with vernalization.

## Results

### Simulation Studies

#### Statistical Power for QTN Detection

**FIGURE 1:**
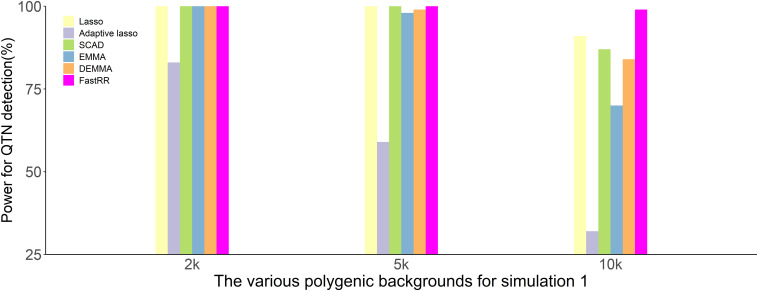
The statistical powers for the fixed position QTN in the first simulation experiment using six methods (lasso, adaptive lasso, SCAD, EMMA, DEMMA, and the FastRR algorithm).

**FIGURE 2:**
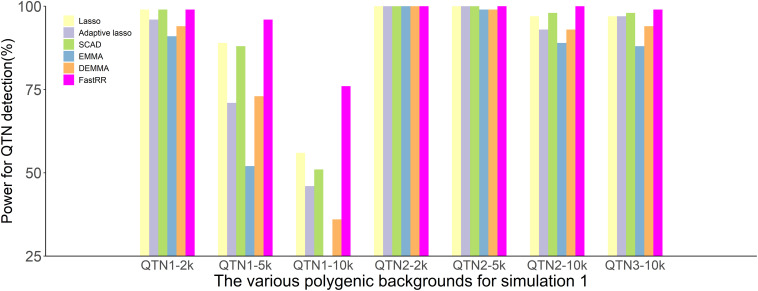
The statistical powers for the minor effect QTNs in the second simulation experiment using six methods (lasso, adaptive lasso, SCAD, EMMA, DEMMA, and the FastRR algorithm).

**FIGURE 3:**
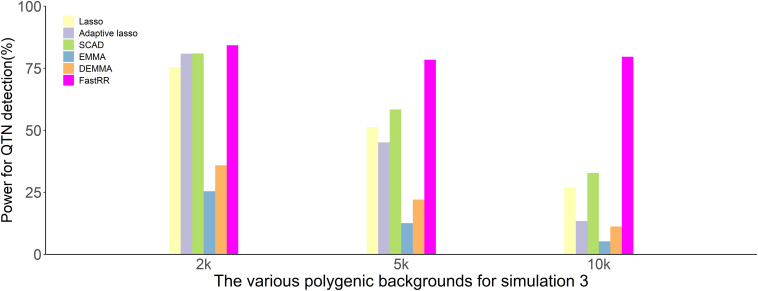
The average statistical powers for all QTNs in the third simulation experiment using six methods (lasso, adaptive lasso, SCAD, EMMA, DEMMA, and the FastRR algorithm).

In the first simulation experiment, only one QTN with a fixed position is simulated, and the power in the detection of the QTN is higher for the FastRR algorithm than for the others (Figure 1 and Table 1). The FastRR algorithm has a dramatically higher statistical power for 10 times polygenic background especially. When five QTNs with the fixed position are simulated in the second experiment, a similar trend is observed (Figure 2 and Tables 2A–C). Three minor effect QTNs (QTL 1 and QTL 2 for three scenarios; QTL 3 for the third scenario) are illustrated in Figure 2, the power of each QTN is less than 100%. Notably, the FastRR algorithm has the highest power for the 98th marker (minor effect locus, *r*^2^ = 2%) under different polygenic backgrounds. One hundred random QTNs are simulated in the third experiment and the total heritabilities are 50%. As the genetic background increases, the power of the FastRR algorithm is getting increasingly high (Figure 3). The results illustrate that the trends are similar to the above experiments (Figure 3). In summary, the FastRR algorithm retains an obviously advantageous performance for the random loci experiment. These results demonstrate the highest power of the FastRR algorithm across all the approaches under various genetic backgrounds.

#### Accuracy for the Estimated QTN Effects

The average effect and mean squared error (MSE) are used to measure the accuracy of an estimated QTN effect. We evaluated the accuracies for the (fixed positions, including simulation experiment 1 and 2) estimates using all six methods (Tables 1, 2A–C). As a result, the estimates for each QTN effect for EMMA, DEMMA, and FastRR are much closer to the true value, and EMMA and DEMMA are slightly better than the FastRR algorithm, nevertheless, EMMA and DEMMA methods have relatively lower power than FastRR. The performance of SCAD, adaptive lasso, and lasso are unsatisfactory. The MSE shows a similar trend to the average effect. On these occasions, the FastRR algorithm, EMMA, and DEMMA methods are recommended for the estimation of QTN effects.

The false positive rate is a crucial index in GWAS. All the false positive rate results of simulation experiment 1 and 2 are listed in Tables 1, 2A–C. Obviously, the false positive rate becomes increasingly high along with the stronger polygenic background. EMMA, DEMMA, and adaptive lasso have a relatively lower false positive rate followed by FastRR, SCAD, and lasso. The false positive rates of all six methods are under control.

#### Computing Time

**FIGURE 4:**
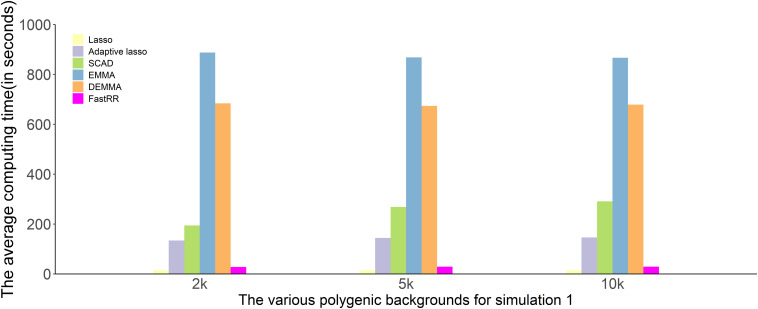
Comparison of computing times to analyze simulation experiment 1 using all six methods (lasso, adaptive lasso, SCAD, EMMA, DEMMA, and the FastRR algorithm).

We compare the computing time of 100 repeated simulated analyses by using six approaches. In each of the three simulation experiments, computing times are recorded and are shown in Figure 4 and Supplementary Figures 1, 2 (Intel Xeon E5-2630 v4, CPU 2.20 GHz, Memory 64G). The computing time of the LASSO and FastRR algorithm have a faster computing speed than the other methods, which are on the same order of magnitude. They are followed by the adaptive lasso and SCAD. DEMMA and EMMA methods take the most expensive computing time at about 600 min, which is nearly seven times more than the FastRR algorithm.

### Analysis of the Rice Data Set

To validate the FastRR algorithm, the grain width trait of rice data is analyzed by using six methods: lasso, adaptive lasso, SCAD, EMMA, DEMMA, and the FastRR algorithm. The rice dataset contains 310,000 SNPs genotyped for 524 inbred varieties. Supplementary Figure 3 shows the LOD plot for three variable selection methods and Manhattan plots for the other three methods. Obviously, DEMMA method and the FastRR algorithm have the identical detected regions, two significant peaks on chromosome 5 and 9. Both DEMMA and FastRR detect the cloned gene *GW5* (Weng et al., 2008) that controls grain width trait. The test statistics of SNP135176 (the most significant SNP) for the DEMMA method and FastRR algorithm are 2.31 × 10^–26^ and 1.92 × 10^–20^, respectively; the *p*-value for the DEMMA method is lower than for the FastRR algorithm. However, the test statistics for the EMMA method do not reach the Bonferroni correction threshold. In addition, three variable selection methods, lasso, adaptive lasso, and SCAD, show unsatisfactory performance according to the LOD scores.

The average computing times are listed in Table 3. The relatively fast methods, lasso, SCAD, and FastRR, are 235.33, 455.31, and 561.31 s, respectively. Lasso is the fastest method among all six methods, which is followed by SCAD and FastRR. In Table 3, the adaptive lasso is different from the above simulation experiments, which consumes much computing time in the cross-validation along with the increasing number of SNPs. The EMMA method takes more than ten times the computing time than the FastRR algorithm.

**TABLE 3:** The computation times (seconds) for analyzing *Arabidopsis* flowering time traits and rice grain width by using lasso, adaptive lasso, SCAD, EMMA, DEMMA, and FastRR methods.

Traits	Lasso	Adaptive lasso	SCAD	EMMA	DEMMA	FastRR
**Rice**						
Grain width	235.33	1067.22	455.31	60813.82	26417.71	561.31
***Arabidopsis***						
LD	36.11	189.36	128.79	1362.55	1117.49	105.17
SD	37.17	159.00	114.17	1350.19	4114.88	112.75
SDV	44.47	140.96	112.34	1665.94	4123.34	107.36

### Analysis of the *Arabidopsis* Data Set

To further validate the FastRR algorithm, this new algorithm FastRR along with lasso, adaptive lasso, SCAD, EMMA, and DEMMA methods are used to reanalyze the *Arabidopsis* data for three traits related to flowering time (LD, SD, and SDV). The results are illustrated in Supplementary Figures 4–6. Each putative QTN (over the threshold) is used to mine the candidate genes by The *Arabidopsis* Information Resource^8^. The FastRR algorithm detects the confirmed genes *AGL*17 and *CDKG*1, which are detected by SCAD and DEMMA as well. From the analysis results, lasso shows several false positive loci in the detection of SD and SDV, meanwhile the adaptive lasso and SCAD methods are inflexible in dissecting the SNPs associated with the target traits. The statistical tests of EMMA are under the Bonferroni corrected threshold. The FastRR algorithm shows a similar pattern as the DEMMA method for all results of three traits, the statistics of part SNPs using the DEMMA method are slightly more significant than the FastRR algorithm, which is similar to the results of the rice datasets.

In terms of the computing speed for all three traits, lasso is computationally much faster than the other methods. The computing times of FastRR, SCAD, and adaptive lasso are on the same order of magnitude, which require less than 200 s. The DEMMA and EMMA methods have much more computational burden than the other methods, both of which require over ten times the computing time required by the FastRR algorithm. Overall, the FastRR algorithm is recommended from the perspective of detection and computing speed across all experiments.

## Discussion

The FastRR algorithm is a multi-stage flexible approach for QTNs dissection in GWAS, and displays high power for detecting QTN of large and minor effects, even under the ten times polygenic background. We aimed to understand the performance of regression analysis methods, thus the following three regression analysis methods, ORR, DRR, and FastRR, are used to analyze simulation experiment 1 and 2. As the results show (Supplementary Tables 1, 2A–C), ORR has the worst detection ability, and even major QTN with large effects are not identified. This explains why ORR is rarely used in GWAS. DRR performs well in simulation 1 and 2, and shows slightly lower power for the major QTNs than FastRR. However, DRR loses power in detecting QTNs with minor effects, and this difference becomes more and more obvious with the increase of the polygenic background. Among three regression analysis methods, the FastRR performs well in the simulation experiment and has the highest statistical power.

Currently, the two-stage methodologies (Tamba et al., 2017; Zhang et al., 2017; Wen et al., 2018) are more popular in GWAS, which are the alternative approaches to solve the “big P, small N” problem. The FASTmrEMMA (Wen et al., 2018; Wen et al., 2020) algorithm is a fast and accurate two-stage methodology for QTNs detection. We further compare the FastRR and FASTmrEMMA algorithm in this study. The results of simulation experiment 1 and 2 are listed in Supplementary Tables 1, 2A–C. Observably, the FastRR and FASTmrEMMA algorithm are powerful in QTNs detection from the perspective of statistical power. However, the estimation of FASTmrEMMA is slightly worse than FastRR, which has a relatively larger MSE. In addition, FASTmrEMMA consumes a median computing time (∼150 s for each replication) among all methods, and much more than FastRR. Therefore, the FastRR algorithm was shown to be a good alternative method for multi-locus GWAS.

Mixed linear model methodologies are mainstream in GWAS; most of them treat QTN effects as fixed effects. In this study, the QTN effects are viewed as random, and it is more consistent with genetic mechanisms (Wen et al., 2018). In order to avoid the influence of the increase of computational complexity, several acceleration techniques have been incorporated into the algorithm. Firstly, we estimate and fix the polygenic-to-residual variance ratio, and then transform the phenotypes and genotypes in the first stage. This technique was adopted in pLARmEB (Zhang et al., 2017) and FASTmrEMMA (Wen et al., 2018), avoiding re-estimating this ratio for each marker. Secondly, the marginal correlation in the second step is similar to the single marker scanning, which quickly filters the unassociated SNPs. The number of SNPs reduces from tens of thousands to hundreds of putative QTNs in the simulation and real data analysis. Thirdly, in the multi-locus model (6), we assume all σγ2=ϕ2, thus only two variance components (**ϕ**^2^ and *σ*^2^) requires DRR to estimate. The results from simulation and real data analysis indicate that the estimation under this simple assumption has achieved better performance for QTN detection and fast computational speed. Lastly, multithreaded marginal correlation is implemented in the FastRR.

Efficient mixed model association and DEMMA as popular single-locus genome scan approaches have been successfully used in GWAS to dissect quantitative traits. However, single-locus approaches ignore the potential information of neighboring markers and fail to consider the joint minor effect of multiple genetic markers on a trait. The FastRR algorithm overcomes this shortcoming. From the results of the simulation, FastRR is more powerful in the detection of QTNs (Figures 2, 3). Although the three popular variable selection approaches, lasso, adaptive lasso, and SCAD, utilize the potential information of markers, the detection and estimation are not accurate (Tables 1, 2A–C). This may be due to the over shrinkage of QTNs, and therefore the effect of QTN is smaller than the true effect; specifically, the minor effect of QTN is shrunk to 0. Consequently, the FastRR algorithm is shown to be more robust in data analysis.

The analysis of large-scale genetic data in GWAS is a hot topic at present. In this study, the correlation coefficients are employed to reduce the dimension of potentially related variables, which are then included in the subsequent multi-locus analysis. The threshold of the correlation coefficient test is set to 0.01 (Tamba et al., 2017), and even the slight correlations between predictors and the response are easily captured. The other thresholds are used, such as 0.001 and 0.0001, which are more rigorous and allows the filtering out of the minor effect loci that will not be included in the multi-locus model. The threshold equal to 0.05 is too loose and includes a large number of SNPs over the threshold; the putative loci are included in the subsequent multi-locus analysis, and furthermore, it is time consuming and results in intractable calculations. Thus, it is reasonable to choose 0.01 as the threshold value in the selection of variables.

## Data Availability Statement

The rice data used for the analysis described in this manuscript was obtained from https://doi.org/10.1093/bioinformatics/btaa345; The Arabidopsis data used for the analysis described in this manuscript was obtained from http://www.arabidopsis.usc.edu/.

## Author Contributions

JZ and JC conceived and supervised the study. JZ, MC, YW, and YL performed all experiments and analyzed the data and revised the manuscript. YZ, MC, SW, and JC made all figures and forms. JZ, YW, and JC also wrote and revised the manuscript. All authors read and approved the final manuscript.

## Conflict of Interest

The authors declare that the research was conducted in the absence of any commercial or financial relationships that could be construed as a potential conflict of interest.

## Footnotes

1 https://cran.r-project.org/web/packages/lars/index.html

2 https://glmnet.stanford.edu/

3 https://cran.r-project.org/web/packages/ncvreg/index.html

4 http://mouse.cs.ucla.edu/emma/

5 https://doi.org/10.1093/bioinformatics/btaa345/

6 https://doi.org/10.1093/bioinformatics/btaa345/

7 http://www.arabidopsis.usc.edu/

8 https://www.arabidopsis.org/

## Abbreviations

MLM: the mixed linear model
GWAS: genome-wide association study
FastRR: fast multi-locus ridge regression
SNP: single nucleotide polymorphism
QTN: quantitative trait nucleotide
EMMA: efficient mixed model association
DRR: deshrinking ridge regression
ORR: ordinary ridge regression
BLUP: best linear unbiased prediction
lasso: least absolute shrinkage and selection operator
SCAD: smoothly clipped absolute deviation
DEMMA: Decontaminated efficient mixed model association
MAF: minor allele frequency
LD: days to flowering under long days
SD: days to flowering under short days
SDV: days to flowering under short days with vernalization
MSE: mean squared error.
