# Supplementary material for: A Fast Multi-Locus Ridge Regression Algorithm for High-Dimensional Genome-Wide Association Studies
Source: Front Genet. 2021 Mar 29;12:649196. doi: 10.3389/fgene.2021.649196 (PMC8041068; doi:10.3389/fgene.2021.649196)
Supplement: Supplementary file 1 [file Data_Sheet_1.pdf]

Supplementary

A Fast Multi-Locus Ridge Regression Algorithm for High-Dimensional Genome-Wide Association Studies

Table S1 Comparison of ORR, DRR and FastRR in the first simulation experiment (three scenarios)

**Polygenic background**	**True value**			**ORR**			**DRR**			**FastRR**			**FASTmrEMMA**		
	**Position**	**Effect**	r^2	**Power (%)**	**Effect (SD)**	**MSE**	**Power (%)**	**Effect (SD)**	**MSE**	**Power (%)**	**Effect (SD)**	**MSE**	**Power (%)**	**Effect (SD)**	**MSE**
**2K**	**98**	**0.7398**	**5%**	0.0	0(0)	0	100.0	0.735(0.091)	0.827	100.0	0.734(0.091)	0.817	100.0	0.702(0.096)	1.053
**5K**	**98**	**0.7398**	**5%**	0.0	0(0)	0	99.0	0.732(0.105)	1.087	100.0	0.729(0.109)	1.188	100.0	0.665(0.123)	2.059
**10K**	**98**	**0.7398**	**5%**	0.0	0(0)	0	82.0	0.768(0.108)	1.004	99.0	0.729(0.131)	1.693	95.0	0.642(0.157)	3.223

Table S2A Comparison of ORR, DRR and FastRR in the second simulation experiment (scenarios 1: two times of polygenic background)

Polygenic background (2K)

QTN	True value			ORR			DRR			FastRR			FASTmrEMMA		
	**Position**	**Effect**	r^2	**Power (%)**	**Effect (SD)**	**MSE**	**Power (%)**	**Effect (SD)**	**MSE**	**Power (%)**	**Effect (SD)**	**MSE**	**Power (%)**	**Effect (SD)**	**MSE**
**1**	**98**	**0.5451**	**2%**	0.0	0(0)	0	93.0	0.597(0.088)	0.959	99.0	0.587(0.094)	1.035	99.0	0.504(0.099)	1.136
**2**	**301**	**0.8622**	**5%**	0.0	0(0)	0	100.0	0.820(0.094)	1.054	100.0	0.820(0.094)	1.054	100.0	0.805(0.104)	1.409
**3**	**540**	**0.8598**	**5%**	0.0	0(0)	0	100.0	0.850(0.089)	0.788	100.0	0.850(0.089)	0.788	100.0	0.828(0.097)	1.030
**4**	**801**	**1.0789**	**8%**	6.0	0.173(0.008)	4.930	100.0	1.059(0.094)	0.911	100.0	1.059(0.094)	0.911	100.0	1.034(0.101)	1.214
**5**	**1000**	**1.2093**	**10%**	35.0	0.171(0.012)	37.711	100.0	1.220(0.094)	0.878	100.0	1.220(0.094)	0.878	100.0	1.184(0.097)	0.996

Table S2B Comparison of ORR, DRR and FastRR in the second simulation experiment (scenarios 2: five times of polygenic background)

Polygenic background (5K)

QTN	True value			ORR			DRR			FastRR			FASTmrEMMA		
	**Position**	**Effect**	r^2	**Power (%)**	**Effect (SD)**	**MSE**	**Power (%)**	**Effect (SD)**	**MSE**	**Power (%)**	**Effect (SD)**	**MSE**	**Power (%)**	**Effect (SD)**	**MSE**
**1**	**98**	**0.5451**	**2%**	0.0	0(0)	0	69.0	0.627(0.080)	0.899	96.0	0.587(0.095)	1.029	89.0	0.482(0.105)	1.317
**2**	**301**	**0.8622**	**5%**	0.0	0(0)	0	99.0	0.839(0.105)	1.138	100.0	0.820(0.126)	1.283	100.0	0.801(0.128)	1.999
**3**	**540**	**0.8598**	**5%**	0.0	0(0)	0	100.0	0.826(0.109)	1.299	100.0	0.826(0.109)	1.299	100.0	0.786(0.123)	2.040
**4**	**801**	**1.0789**	**8%**	1.0	0.244(0.000)	0.697	100.0	1.075(0.116)	1.334	100.0	1.075(0.116)	1.334	100.0	1.034(0.119)	1.606
**5**	**1000**	**1.2093**	**10%**	20.0	0.220(0.016)	19.588	100.0	1.232(0.100)	1.049	100.0	1.232(0.100)	1.049	100.0	1.176(0.114)	1.394

Table S2C Comparison of ORR, DRR and FastRR in the second simulation experiment (scenarios 3: ten times of polygenic background)

Polygenic background (10K)

QTN	True value			ORR			DRR			FastRR			FASTmrEMMA		
	Position	Effect	r^2	Power (%)	Effect (SD)	MSE	Power (%)	Effect (SD)	MSE	Power (%)	Effect (SD)	MSE	Power (%)	Effect (SD)	MSE
**1**	**98**	**0.5451**	**2%**	0.0	0(0)	0.000	36.0	0.706(0.069)	1.092	76.0	0.644(0.095)	1.160	55.0	0.524(0.103)	0.595
**2**	**301**	**0.8622**	**5%**	0.0	0(0)	0.000	92.0	0.852(0.106)	1.033	100.0	0.830(0.126)	1.668	99.0	0.762(0.146)	3.074
**3**	**540**	**0.8598**	**5%**	0.0	0(0)	0.000	94.0	0.857(0.128)	1.529	99.0	0.842(0.140)	1.960	99.0	0.784(0.157)	2.978
**4**	**801**	**1.0789**	**8%**	0.0	0(0)	0.000	100.0	1.083(0.141)	1.958	100.0	1.083(0.141)	1.958	100.0	1.024(0.154)	2.632
**5**	**1000**	**1.2093**	**10%**	4.0	0.280(0.009)	3.453	100.0	1.204(0.152)	2.290	100.0	1.204(0.152)	2.290	100.0	1.129(0.164)	3.305

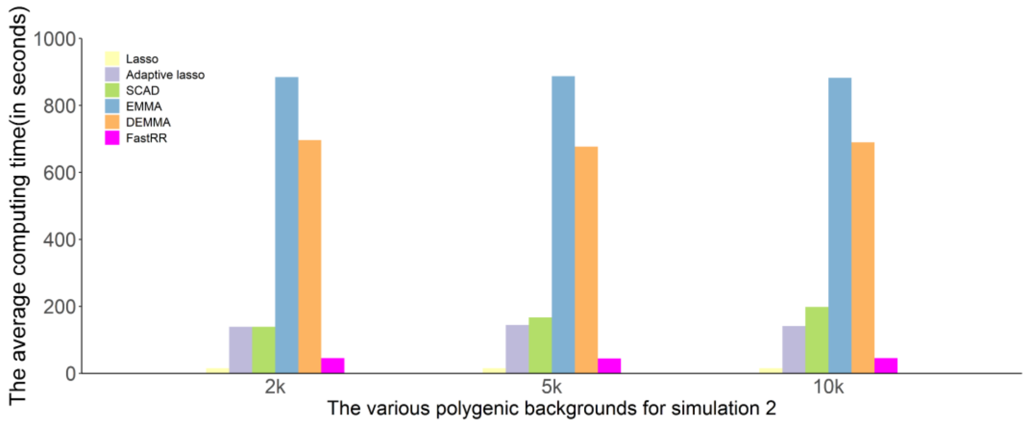

Figure S1 Comparison of computing times to analyze simulation experiment 2 using all six methods (lasso, adaptive lasso, SCAD, EMMA, DEMMA, and the FastRR algorithm).

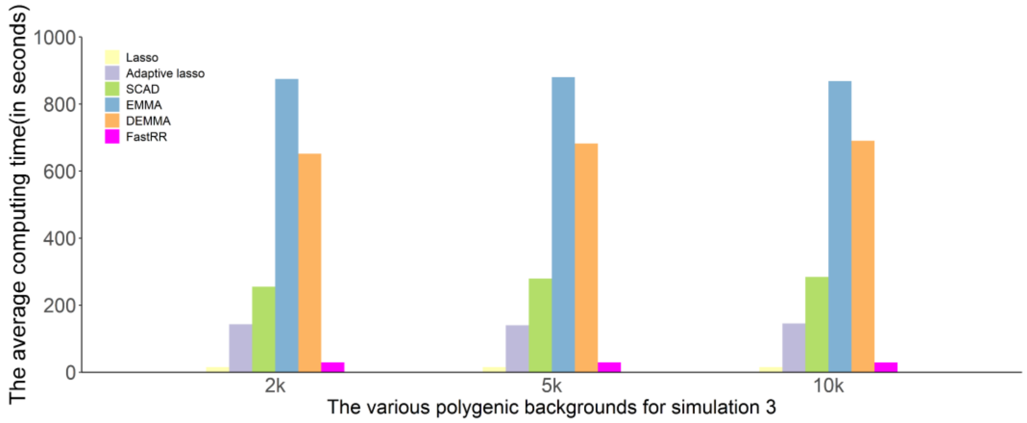

Figure S2 Comparison of computing times to analyze simulation experiment 3 using all six methods (lasso, adaptive lasso, SCAD, EMMA, DEMMA, and the FastRR algorithm).

A

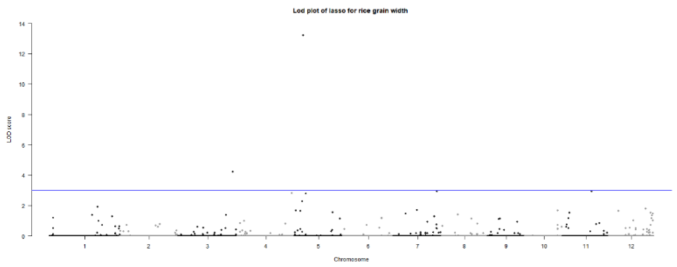

B

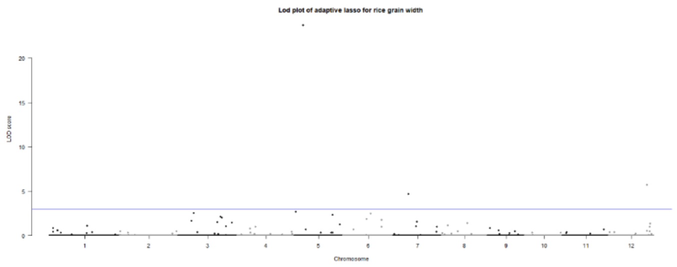

C

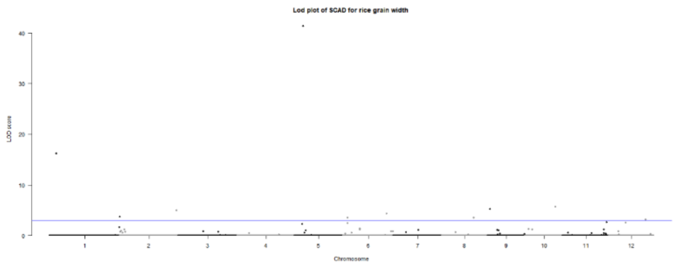

D

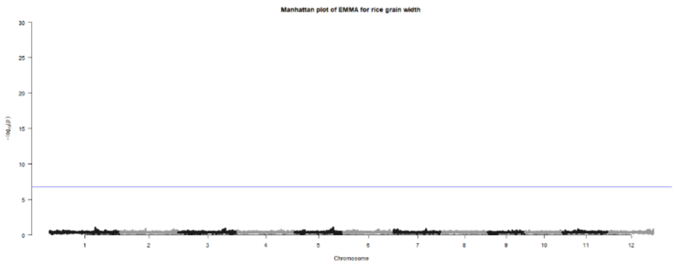

E

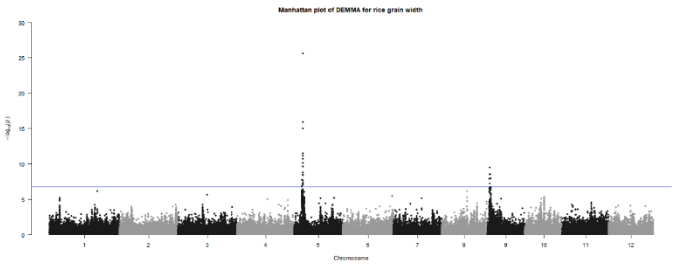

F

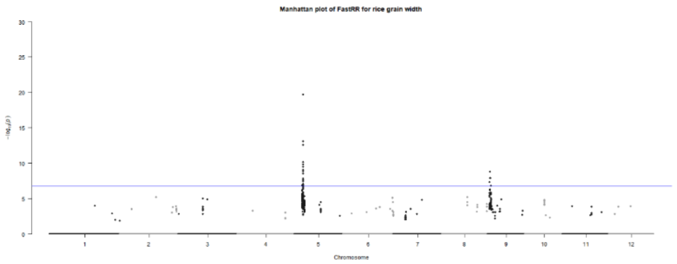

Figure S3 LOD plots of rice grain width from lasso (A), adaptive lasso (B) and SCAD(C) and Manhattan plots from EMMA (D), DEMMA (E) and FastRR (F).

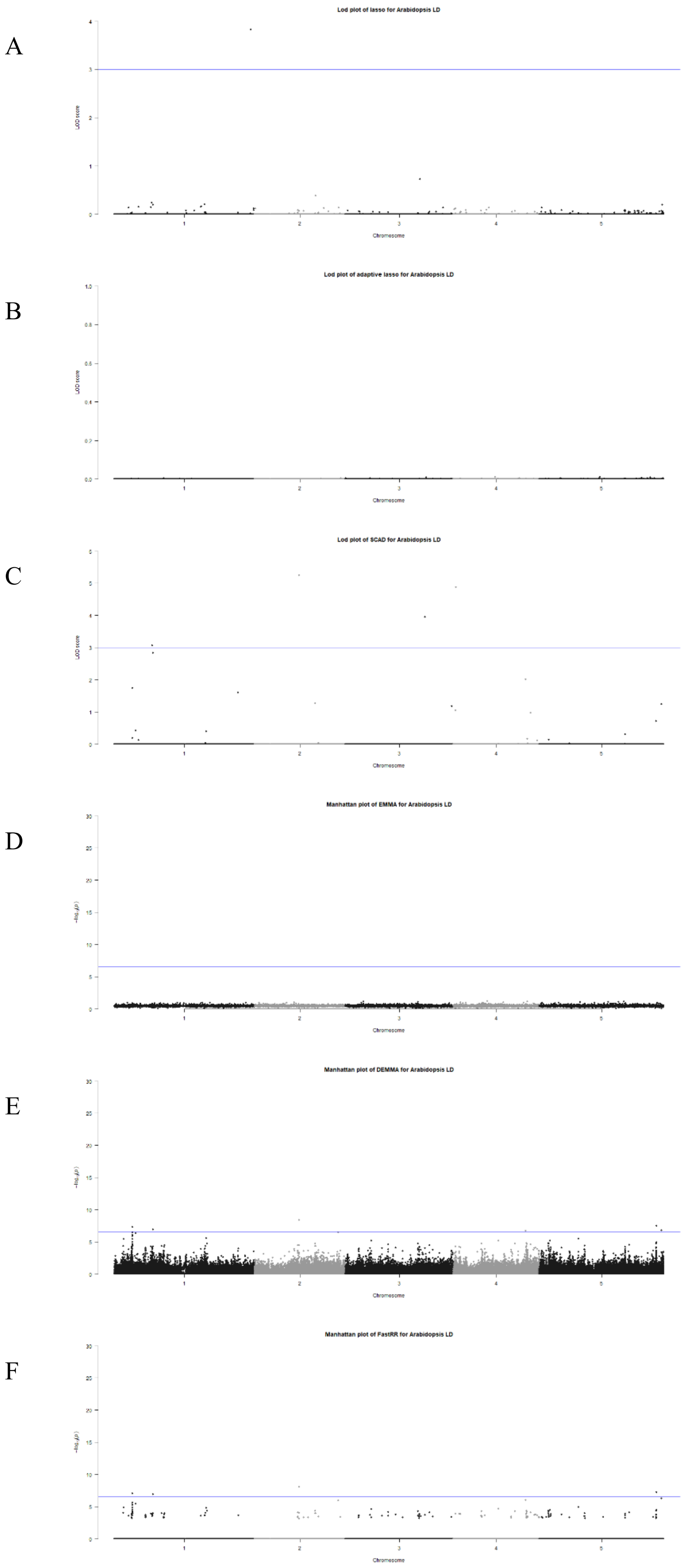

Figure S4 LOD plots of *Arabidopsis* LD from lasso (A), adaptive lasso (B) and SCAD(C) and Manhattan plots from EMMA (D), DEMMA (E) and FastRR (F).

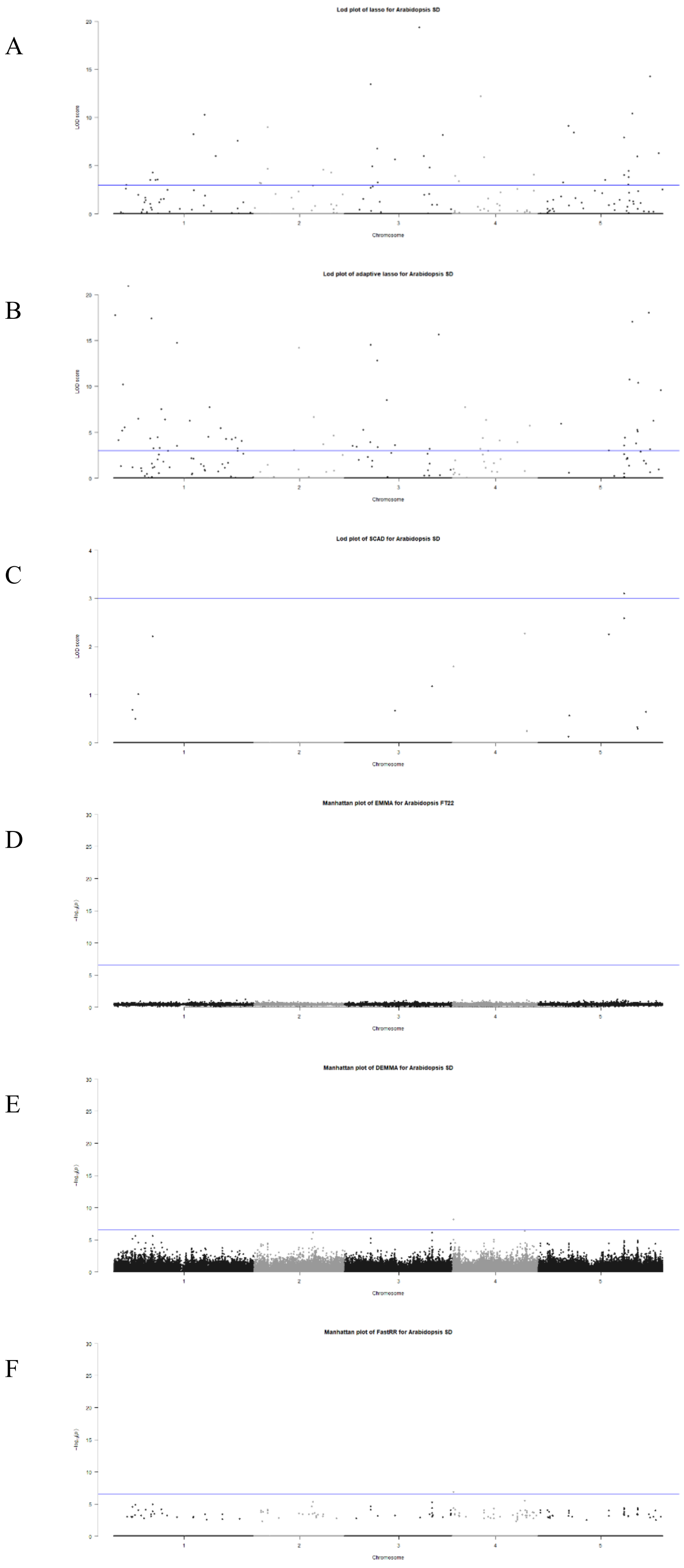

Figure S5 LOD plots of *Arabidopsis* SD from lasso (A), adaptive lasso (B) and SCAD(C) and Manhattan plots from EMMA (D), DEMMA (E) and FastRR (F).

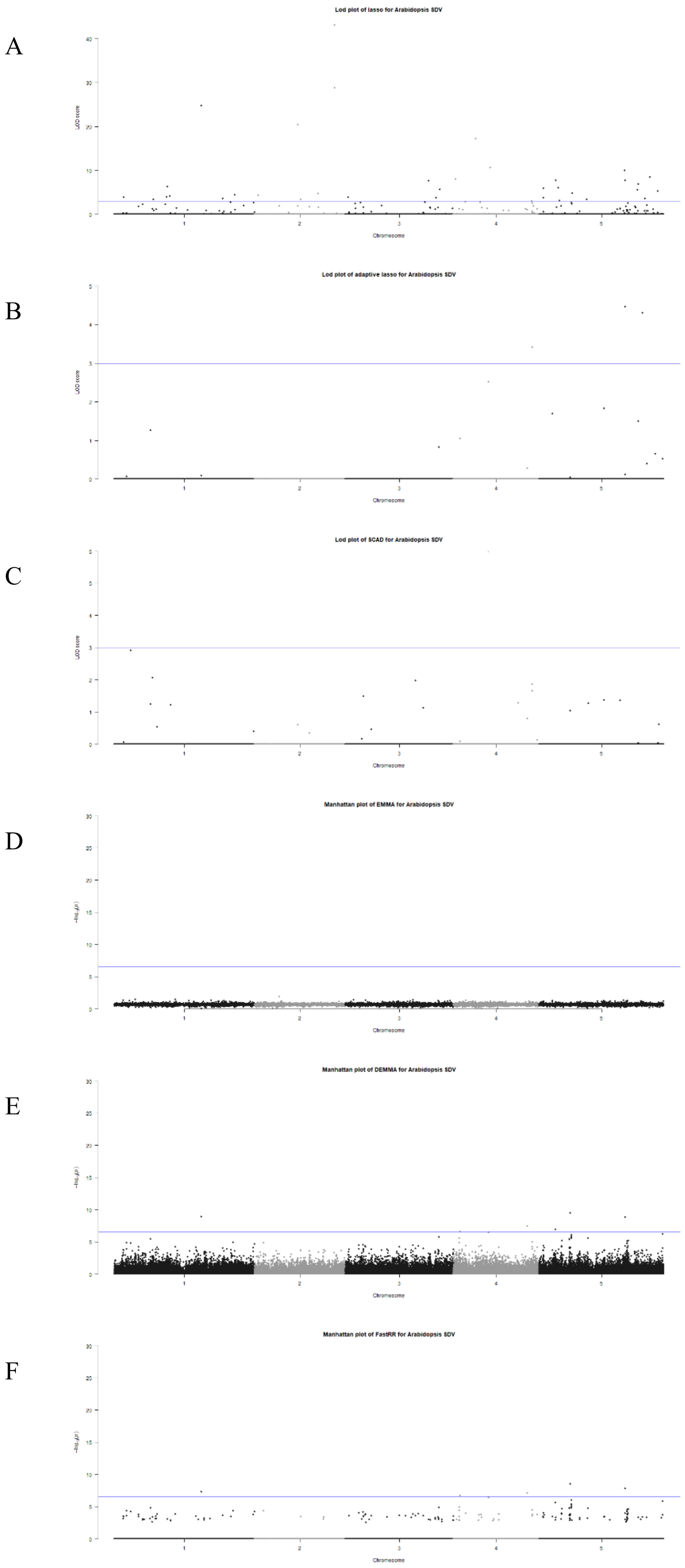

Figure S6 LOD plots of *Arabidopsis* SDV from lasso (A), adaptive lasso (B) and SCAD(C) and Manhattan plots from EMMA (D), DEMMA (E) and FastRR (F).
